# Drug Delivery Across the Blood–Brain Barrier: A New Strategy for the Treatment of Neurological Diseases

**DOI:** 10.3390/pharmaceutics16121611

**Published:** 2024-12-19

**Authors:** Yimai Jiao, Luosen Yang, Rujuan Wang, Guoqiang Song, Jingxuan Fu, Jinping Wang, Na Gao, Hui Wang

**Affiliations:** 1 Key Laboratory of Molecular Biophysics, Institute of Biophysics, School of Health Sciences and Biomedical Engineering, Hebei University of Technology, Tianjin 300401, China; 19943563157@163.com (Y.J.); 18632809221@163.com (R.W.); songguoqiangx@163.com (G.S.); sophiefjx@outlook.com (J.F.); wangjp@hebut.edu.cn (J.W.); 2 School of Chemical Engineering and Technology, Hebei University of Technology, Tianjin 300401, China; 15533755304@163.com; 3 Tianjin’s Clinical Research Center for Cancer, Key Laboratory of Cancer Prevention and Therapy, Tianjin, National Clinical Research Center for Cancer, Tianjin Medical University Cancer Institute & Hospital, Tianjin 300060, China

**Keywords:** blood–brain barrier, central nervous system, drug delivery, neurological disease, nanotechnology

## Abstract

The blood–brain barrier (BBB) serves as a highly selective barrier between the blood and the central nervous system (CNS), and its main function is to protect the brain from foreign substances. This physiological property plays a crucial role in maintaining CNS homeostasis, but at the same time greatly limits the delivery of drug molecules to the CNS, thus posing a major challenge for the treatment of neurological diseases. Given that the high incidence and low cure rate of neurological diseases have become a global public health problem, the development of effective BBB penetration technologies is important for enhancing the efficiency of CNS drug delivery, reducing systemic toxicity, and improving the therapeutic outcomes of neurological diseases. This review describes the physiological and pathological properties of the BBB, as well as the current challenges of trans-BBB drug delivery, detailing the structural basis of the BBB and its role in CNS protection. Secondly, this paper reviews the drug delivery strategies for the BBB in recent years, including physical, biological and chemical approaches, as well as nanoparticle-based delivery technologies, and provides a comprehensive assessment of the effectiveness, advantages and limitations of these delivery strategies. It is hoped that the review in this paper will provide valuable references and inspiration for future researchers in therapeutic studies of neurological diseases.

## 1. Introduction

The blood–brain barrier (BBB) is a highly selective barrier located between the endothelial cells of the cerebral vasculature, which consists mainly of tightly connected cells [1]. This barrier effectively isolates substances in the bloodstream from brain tissue, preventing direct contact and thus protecting the central nervous system (CNS) from potentially harmful substances [2,3]. The BBB plays an important role in the protection of the CNS and the maintenance of its homeostasis. However, dysfunction of the BBB has been associated with a variety of neurological disorders. A comprehensive investigation of the mechanisms underlying the BBB and its dynamics during disease progression may facilitate the development of novel therapeutic strategies. These strategies may include the design of new drugs, immune interventions, and cellular therapies, with the aim of increasing the level of control of these complex diseases and ultimately improving the quality of life of patients. Infection, trauma and other pathological states may result in the compromise of the structure and function of the BBB, leading to increased permeability and elevated levels of neuroinflammation. For example, in the case of meningitis and multiple sclerosis (MS), damage to the BBB allows immune cells to invade the CNS [4]. In brain tumors (e.g., gliomas), tumor cells can disrupt the integrity of the BBB, allowing larger molecular weights and more cells to enter the brain [5]. This is thought to be a key mechanism in neurodegenerative diseases, such as Alzheimer’s disease (AD) and Parkinson’s disease (PD), where the impaired function of the BBB is believed to be closely related to the pathological progression of the disease [6,7].

In recent years, research on targeted drug delivery across the BBB has received considerable attention from the scientific community worldwide. Nanocarrier technologies have been the subject of extensive study by researchers with the objective of enhancing the capacity of pharmaceutical agents to traverse the BBB. These carriers can be employed to enhance targeting and biocompatibility through surface modification, alteration of particle size and optimization of release mechanisms [8,9]. For example, some researchers have employed the use of antibodies or ligands to modify nanoparticles with the objective of enhancing their capacity to bind to specific receptors within the brain. This, in turn, has the potential to improve the efficacy of nanoparticles in traversing the BBB [10]. Furthermore, it has been demonstrated that low-intensity focused ultrasound (FUS) can facilitate the transient opening of the BBB, thereby enabling the effective delivery of drugs into the brain. This approach demonstrates considerable promise in the context of brain disease treatment [11]. The chemical structures of certain small molecule drugs have been designed with the objective of enhancing their lipid solubility across the BBB or modifying their polarity to improve their ability to penetrate the BBB. L-DOPA (levodopa) is a drug used to treat Parkinson’s disease, and its lipophilic nature helps it penetrate the BBB and convert it into dopamine in the brain, replenishing the neurotransmitters needed by the patient [12]. Moreover, researchers have developed an array of biodegradable polymers, including polylactic acid (PLA), for the fabrication of targeted drug delivery systems [13]. These carriers are capable of releasing drugs within the brain in a controlled manner, thereby improving the efficacy of the therapeutic agent. A number of studies have concentrated on the utilization of lipid nanoparticles, the modification of polymer nanoparticles to enhance drug targeting, and peptide modification to optimize the binding capacity of the carriers to the BBB receptor [14,15].

Conventional drug delivery methods often encounter difficulties in penetrating the BBB. In contrast, drug delivery across the BBB can enhance the ability of drug molecules to penetrate the BBB through the use of special carriers (e.g., nanoparticles, liposomes, exosomes, etc.) or through specific biological pathways (e.g., receptor-mediated cytotoxicity). The field of trans-BBB targeted drug delivery is experiencing rapid growth on an international scale. Novel therapeutic approaches have the potential to enhance drug targeting, reduce systemic side effects and improve efficacy when compared to traditional drug delivery modalities. This could have a significant impact on the quality of life of patients, particularly those with chronic diseases that require long-term treatment. Nevertheless, numerous challenges persist, including the stability of drug delivery systems, pharmacological evaluation, long-term safety, and clinical translation. It is imperative that research in these areas be further strengthened.

This article presents a review of the drug delivery methods for the BBB that have been developed in recent years. The methods covered include physical, biological and chemical methods, as well as nanoparticle delivery technologies and combinatorial strategies. The discussion focuses on the design principles, biocompatibility and targeting ability of these methods. Furthermore, the feasibility, advantages and disadvantages of these approaches are analysed with the inclusion of pertinent examples. In conclusion, the article presents a synthesis of the aforementioned strategies, elucidating their significance and advantages. It aspires to serve as a reference point and a source of guidance for future research endeavors in the domain of neurological disorders, while also facilitating the advancement of research in related fields.

## 2. Physiological and Pathological Characteristics of the BBB

**Figure 1:**
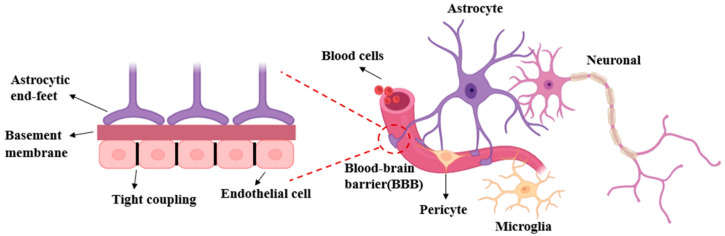
Diagram of the structural components of the blood–brain barrier.

The BBB is a complex physiological structure, primarily comprising brain capillary endothelial cells, basement membranes, pericytes, and glial cells (e.g., astrocytes) [16]. Its physiological structure is illustrated in Figure 1. The primary function of the BBB is to safeguard the CNS from detrimental substances in the external environment, while maintaining the homeostasis of the intracerebral environment.

Endothelial cells play a pivotal role in the BBB, with primary responsibility for the structure and function of the barrier. Endothelial cells are connected to each other by tight junctions, which prevent macromolecules and a wide range of water-soluble substances from freely traversing the BBB, thereby forming a selectively permeable barrier. The presence of tight junctions ensures that only a limited number of substances are able to pass through the BBB, thereby protecting the CNS from harmful substances and pathogens while maintaining a stable state of the intracerebral environment [17,18]. Moreover, endothelial cells exhibit selective permeability to specific nutrients (e.g., glucose and amino acids) and small molecules (e.g., oxygen and carbon dioxide) [19]. Specific transporter proteins are located on the surface of endothelial cells, facilitating the transmembrane transport of essential substances. For instance, glucose transporter protein (GLUT1) and amino acid transporter proteins efficiently deliver nutrients required by brain cells [20,21]. Additionally, they synthesize and secrete cytokines and signaling molecules that can influence surrounding glial cells, immune cells and other neurons, thereby modulating the function of the BBB [22]. Furthermore, endothelial cells play a role in regulating neuroinflammatory responses. In the context of neurological diseases or pathologies, endothelial cells are capable of responding to inflammatory signals by modifying intercellular connectivity and increasing barrier permeability. This allows the entry of immune cells or drug treatment. Such alterations assist in combating pathogens, yet they may also precipitate pathological damage [23]. Endothelial cells interact with glial cells and neurons, which communicate with each other through secreted information molecules and specific receptors to regulate the physiological state and responsiveness of the BBB. This communication is of particular importance in the context of pathology and the adaptation to various physiological changes [24,25]. Nevertheless, in diverse neurological disorders, the functionality of endothelial cells may be impaired, which can result in anomalous BBB permeability and, subsequently, influence CNS function.

The basement membrane plays a role in maintaining the structural integrity and functionality of the BBB. The basement membrane represents a distinct form of extracellular matrix (ECM) predominantly situated beneath endothelial and epithelial cells. The basement membrane provides structural support for endothelial cells in the BBB and participates in the homeostatic maintenance and repair of the barrier by regulating substance permeability and facilitating cell signaling and cellular interaction. The basement membrane provides a supportive framework for the endothelial cells in the BBB, thereby maintaining the morphology and relative position of the endothelial cells and thus capillary stability [26,27,28].The basement membrane comprises a variety of biochemical components that can regulate the exchange of substances between the blood and brain tissue. The components of the basement membrane, including collagen, glycoproteins and glycosaminoglycans, can influence the selectivity and permeability of substances through the BBB. Furthermore, the basement membrane contains a variety of proteins and polysaccharide compositions that are involved in intercellular signaling through interactions with endothelial cell surface receptors [29]. This signaling has been demonstrated to influence the behaviour of endothelial cells, e.g., by enhancing or attenuating barrier permeability during inflammatory processes or other physiological states. Furthermore, the basement membrane serves as a crucial mediator of interactions between endothelial cells and surrounding cells (e.g., astrocytes, microglia, etc.). It provides a shared environment for these cells, thereby contributing to the regulation and maintenance of the overall function of the BBB.

Glial cells are essential for the formation, maintenance and regulation of the BBB. In addition to providing structural support, they participate in the immune response and repair process by regulating intercellular signaling and nutrient transport. The glial cells present in the BBB are primarily astrocytes and other types of glial cells, including oligodendrocytes and microglia [30]. Astrocytes assist in maintaining the integrity of the BBB by forming a tight wrapping around the brain’s blood vessels through their extended peduncles. The peduncles are capable of engaging in interactions with endothelial cells and facilitating the formation of tight junctions. Glial cells, particularly astrocytes, augment the functionality of the BBB by secreting an array of neurotrophic factors that stimulate the growth and maturation of endothelial cells. Furthermore, astrocytes utilise their own released signaling molecules (e.g., peptides and growth factors) to regulate tight junctions in endothelial cells. In the BBB, glial cells are responsible for regulating the ionic concentration of the intracerebral environment and maintaining neurotransmitter homeostasis through the action of transporter proteins, thereby protecting neurons. Furthermore, astrocytes guarantee that brain cells receive an adequate supply of energy and maintain ionic and nutritional homeostasis by transporting glucose and other nutrients [31]. Microglia, the primary immune cells of the CNS, possess the capacity to monitor and eliminate pathogens. In the event of damage to the BBB or the presence of an infection, microglia are activated, undergoing morphological changes and releasing cytokines in response to the ensuing inflammatory response [32]. Following nerve injury, glial cells are capable of accumulating at the site of injury and releasing growth factors and inflammatory mediators, which facilitate the repair and regeneration of the injured tissue. The proliferation and migration of glial cells contribute to the formation of the nascent BBB and the restoration of its function. Glial cells regulate nerve signals and BBB permeability through their participation in signaling interactions between neurons, glial cells, and endothelial cells [33]. These interactions guarantee the efficient transmission of information and enable adaptive responses.

The BBB is not only distinctive in its anatomical structure, but also plays a role via its physiological function. The structural characteristics of the BBB make it an exceptionally efficient screening system, capable of effectively safeguarding the CNS from external noxious substances while simultaneously providing essential nutrient support to nerve cells. The mechanisms of action of the BBB include strict regulation of substance permeability, complex transport mechanisms, and responsiveness to external changes. The BBB is responsible for regulating substance permeability and, due to its highly selective permeability, it effectively prevents the entry of most macromolecules and water-soluble substances in the bloodstream into the CNS. At the same time, the BBB allows certain small molecules (including oxygen, carbon dioxide, etc.) to be transported, either by simple diffusion or by specific transport mechanisms. The latter include active transport, receptor-mediated transport, and the diffusion of lipids and small molecules. Active transport, for example, involves the active transportation of glucose to the CNS via specific transporter proteins (GLUTs), which enables the brain to meet its metabolic needs. In contrast, receptor-mediated transport involves the crossing of nerve growth factor (NGF) or transferrin through the BBB via its specific receptor. The BBB serves to protect the CNS by preventing the indiscriminate passage of pathogens, toxins, and the majority of drugs. Furthermore, it ensures the timely delivery of essential nutrients to brain tissue via a selective transport mechanism. Additionally, cells within the BBB are capable of sensing changes in the external microenvironment and regulating their own permeability. For example, in conditions characterized by an inflammatory response, the BBB may undergo functional changes in response to immune signals [34]. Furthermore, astrocytes and other glial cells play a pivotal role in regulating the internal environment, including pH and electrolyte concentration factors.

It is hypothesized that the dysfunction of the BBB is closely associated with the onset and progression of neurological disorders, including neuroinflammation, brain tumors and neurodegenerative diseases. In diseases such as MS, AD and PD, the integrity of the BBB affects the health of neuronal cells [18,35]. Gliomas and brain metastases are two common types of brain tumor that are characterized by disruption of the BBB structure, resulting in impaired integrity of the BBB and increased vascular permeability, forming the so-called blood–brain tumor barrier (BTB). This change not only facilitates the entry of tumor cells and their secretions into brain tissue, accelerating tumor growth, but also complicates the treatment process, as they need to overcome BBB barriers for effective drug delivery [36]. Conversely, in cerebrovascular diseases, such as stroke and ischemic disease, disruption of the BBB may result in brain oedema, cell death and neurological impairment [37].

## 3. Drug Targeted Delivery Challenges

As a consequence of the ageing of society and increasing competitive pressure, CNS diseases have become the second most common type of disease. Nevertheless, the success rate of CNS drug development is only 8 per cent, in comparison with the success rate of approximately 20 per cent for cardiovascular drugs [38]. A significant factor influencing the efficacy of CNS drug development is the BBB, because it hinders the passage of many large molecule drugs and small molecule drugs [39].

The primary challenges to delivery of drug molecules through the BBB are the structural properties and selective permeability of the BBB itself. Due to the lipid bilayer structure of the BBB, lipid-soluble drugs are more readily permeable, whereas water-soluble drugs face a greater challenge [19]. The specific substrate specificity of transporter proteins and the limited number of loaded drugs restrict their potential for entering the CNS through the transporter mechanism. The molecular weight, polarity and hydrophobicity of many drugs (such as Oxycodone) are not compatible with the requirements for penetration of the BBB, so this may result in rejection or degradation of the drug within the BBB [38,40].

Furthermore, deficiencies in biological modelling and assessment techniques present additional challenges for drug-targeted transport. For instance, the current in vitro and in vivo models may not accurately reflect the intricacies of the human BBB, which introduces uncertainty in the preclinical testing of drugs. Vitro BBB models are unable to fully reproduce the complex microenvironment and dynamics of the BBB in vivo. For instance, a considerable number of in vitro models lack the involvement of crucial cell types. Additionally, in vitro models may be incapable of fully simulating the mechanisms of drug metabolism, transport, and removal in vivo [41,42]. This increases the risk of preclinical testing. With regard to safety and side effects, alterations in BBB permeability may result in the influx of noxious substances, immune cells, and other drugs into the CNS, with the potential for adverse effects and reactions. For instance, the utilization of focused ultrasound (FUS) in conjunction with microbubble technology to transiently permeabilize the BBB in a defined region in order to facilitate drug penetration may result in the irreversible or reversible impairment of the BBB, which may not be appropriate for all patients [43].

Furthermore, an individual’s age, sex, genetics, and physiological status (e.g., pregnancy status) may influence the function of the BBB and the capacity of drugs to penetrate the brain. The functionality of the BBB may vary in individuals of different age groups. To illustrate, the BBB is not fully developed in children, whereas older individuals may exhibit increased BBB permeability and decreased function associated with neurodegenerative diseases [44]. It is possible that gender differences may have a significant impact on drug metabolism, feedback regulation, and drug response, particularly in certain diseases (e.g., AD and PD), where gender differences are manifested in disease progression and drug response [45]. Furthermore, genetic variability between individuals can influence the structure, function, and response of the BBB to drugs. As a consequence of these factors, the efficacy of a drug in the preclinical phase may diverge considerably from that observed in the clinical phase, thereby increasing uncertainty and elevating the risk of failure in clinical trials. Individual differences further complicate the determination of optimal drug dosage and dosing strategies, underscoring the necessity for individualized medicine.

Neurological disorders affect the central and peripheral nervous systems. They may impair the function, structure or health of neurons, which in turn affects the body’s motor abilities, sensation, cognition and autonomic functions [46]. In certain pathological conditions, the BBB may be compromised, which can alter the manner in which drugs enter the brain and the extent to which they do so. This may also result in the rejection of molecules or cells that should be in close proximity. Such alterations not only impact the efficacy of the therapeutic agent but may also elevate the probability of adverse effects.

AD is a progressive neurodegenerative disease and the most common form of dementia [47]. The disease primarily affects the ability to remember, think and behave [48], and may ultimately affect all aspects of daily life. In patients with AD, the permeability of the BBB may be increased, resulting in the abnormal passage of plasma components (e.g., macromolecular proteins), which may affect the stability of the intracerebral environment. The deposition of amyloid may result in functional impairment of endothelial cells due to inflammatory responses. Local inflammation may also facilitate the rejection of certain immune cells and large molecule drugs, thereby hindering the efficacy of therapeutic interventions and compromising the barrier function of the BBB [49]. The altered permeability allows certain drugs (e.g., anti-Alzheimer drugs) to enter the brain tissue with greater ease, but this may also pose a risk of toxicity.

PD is a chronic neurodegenerative disease that is characterized by tremor, muscle stiffness, slowed movements and postural instability [50]. The loss of dopaminergic neurons causes damage to the BBB in patients with PD. This damage is caused by inflammatory processes, which result in alterations to the permeability of the BBB and a reduction in endothelial cell activity [51]. As the disease progresses, a decline in the functionality of endothelial cells may cause a decrease in the body’s ability to resist neurotoxins.

A brain tumor is defined as a cluster of abnormal cells that form within the brain tissue. These tumors may be classified as either benign or malignant, with the specific symptoms varying according to the location and size of the tumor. These symptoms may include headaches, seizures, and alterations in cognitive function. Brain tumors can be classified into two main categories: primary brain tumors, which include conditions such as glioblastoma, and metastatic brain tumors [52]. Brain tumors are typically accompanied by a significant disruption of the BBB, which may manifest as structurally incomplete and with highly permeable new vessels. The destruction of the BBB may facilitate the entry of chemotherapeutic and targeted drugs into the tumor area, although this process may also result in damage to normal brain tissue [53]. Given the compromised functionality of the BBB, it is possible that not only may drugs gain access to the tumor area, but they may also impact normal brain cells, resulting in significant adverse effects.

MS is an autoimmune disease that causes the destruction of myelin in the CNS. The symptoms of this disease include blurred vision, limb weakness, sensory disturbances, and motor incoordination [54]. The immune system initiates an attack on the body’s neural tissues, resulting in the disruption of nerve signaling. In MS, an immune-mediated response results in the dysfunction of the BBB and increased permeability. Demyelination injury affects BBB integrity and leads to neurodegenerative changes [55]. Therefore, drug delivery needs to be adjusted according to the changes that occur after BBB injury.

In these diseases, the integrity and function of the BBB is compromised, which not only impedes effective drug penetration but may also interfere with normal physiological functions. It is important to develop targeted drug delivery strategies. Although ongoing scientific research is addressing the challenges of BBB-targeted drug delivery, a deeper understanding of the biological nature and dynamics of BBB is needed to optimize the efficiency of drug delivery and ensure the safety of treatments.

## 4. New Strategies for the Treatment of Neurological Diseases

In recent times, researchers from a multitude of global research institutions have conducted comprehensive studies on drug delivery strategies across the BBB. The findings of these studies have led to the proposal of a series of innovative tools and strategies, which are presented in this paper in a systematic manner.

### 4.1. Physical Method

#### 4.1.1. Ultrasonic Open BBB

The application of ultrasound is not limited to the fields of diagnosis and fragmentation; it also shows potential in the treatment of diseases. The utilization of focused ultrasound technology is predicated upon the principle of acoustic wave propagation, whereby a focal point is created through a concave transducer, thereby facilitating the localised opening of the BBB. This process entails the concentration of energy at the focal point, which then produces a mechanical or thermal effect, or a combination of both [56]. By controlling the frequency and other conditions, it is possible to open the BBB in a targeted manner, without damaging the surrounding tissues and organs, thus achieving targeted drug delivery. Nevertheless, the opening of the BBB through focused ultrasound remains an inherently unstable process. As early as 1981, Vykhodtseva had already explored the potential of ultrasound parameters for producing small and controlled lesions, but her efforts were unsuccessful. In the years that followed, numerous scientists endeavored to investigate the potential of focused ultrasound techniques to effectively open the BBB. However, the results of these endeavors were, at the time, deemed unsatisfactory [57,58]. In 2001, Hynynen et al. reported the first successful instance of the successful opening of the blood–brain barrier using low-intensity focused ultrasound after intravenous injection of an ultrasound contrast agent [59].This approach has the additional benefit of avoiding cranial overheating while causing essentially no damage to surrounding tissues. The drug can be wrapped with contrast agent microbubbles using the drug transport properties of ultrasound microbubble contrast agents. This method has several advantages. Firstly, it prevents the degradation and inactivation of the drug in the process of body circulation. Secondly, it reduces systemic toxicity and side-effects. Thirdly, it enables the drug to be released in a specific area through ultrasound-targeted microbubble-breaking technology. It has been demonstrated that the extent of BBB opening is contingent upon a number of factors, including acoustic frequency, ultrasound intensity, pulse length, pulse repetition frequency (PRF) and acoustic duration. Additionally, the size, dose and type of microbubbles have also been identified as influencing variables [60].

It is currently accepted within the scientific community that the mechanism by which ultrasound combined with microbubbles opens the BBB is primarily due to the cavitation effect and the mechanical effect. The principal mechanism through which the cavitation effect exerts its influence on the BBB is the transient opening of tight junctions and the transient increase in cell membrane permeability. The propagation of ultrasound through tissue results in alternating compression and expansion of tissue molecules, which in turn leads to the formation of microbubbles. In specific ultrasound conditions, the microbubbles are in a stable state of compression and expansion. Should the ultrasound conditions continue to be increased, the microbubbles will rapidly expand and rupture, generating a substantial amount of energy in the surrounding microenvironment. The introduction of ultrasound microbubbles markedly enhances the efficiency of the cavitation effect, due to the substantial number of microbubbles entering the circulation as cavitation nuclei, thereby increasing the number of cavitation nuclei per unit volume. Consequently, the ultrasound energy necessary to elicit an equivalent cavitation effect is reduced by two orders of magnitude in comparison to the use of ultrasound alone. Furthermore, the utilization of ultrasound microbubble technology has been demonstrated to enhance the permeability of the BBB through two principal mechanisms: circumferential pressure and shear stress. The other mechanism of opening is the mechanical effect, which involves the microbubbles being pressed against the vessel wall by the radial pressure of the ultrasound waves. This causes microbubbles to act on the vessel wall, resulting in either a cavitation effect or a thermal effect. This process can elucidate the relationship between pulse duration and the degree of BBB opening. It can be posited that, under identical circumstances, the degree of BBB opening increases with increasing pulse duration within a specified time frame [61,62,63].

The combination of ultrasound and microbubble technology has been proved to be an effective treatment for a range of neurological disorders. In a two-phase clinical trial conducted by Mehta and colleagues, the results of the trial demonstrated that cognitive performance improved in all five patients following treatment, and no damage was observed to brain tissue during the process. Furthermore, no significant intracranial hemorrhage or other serious adverse effects were observed in any of the participants [64]. In a similar vein, Arrieta et al. employed this technique to open the BBB in glioblastoma (GBM) patients and mice, which improved the therapeutic effect [65]. Wang et al. enhanced the therapeutic efficacy of the treatment by preparing a loading of brain-derived nerve growth factor (BDNF) retrovirus (MpLXSN-BDNF) microbubbles and combining them with ultrasound to open the BBB of rats suffering from AD for treatment [66]. This disruption of the BBB is transient, with the BBB’s integrity rapidly restored following the cessation of sound waves, and complete closure occurring within 6 to 24 h [63]. In conclusion, the utilization of ultrasound in conjunction with microbubble technology to breach the BBB for the purpose of targeted drug delivery in the treatment of neurological disorders is a safe and viable approach.

#### 4.1.2. Magnetic Targeted Delivery

**Figure 2:**
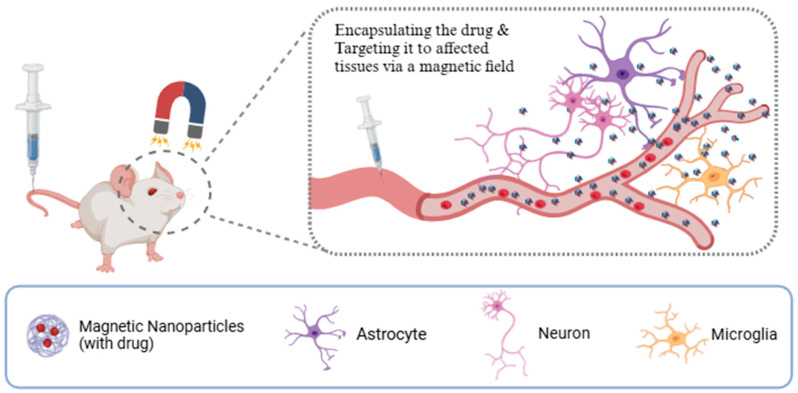
Schematic representation of magnetic NPs functioning under the effect of external magnetic field.

In addition to using ultrasound to facilitate the opening of BBBs, the inherent properties of magnetic nanoparticles (MNPs) can be harnessed to achieve a breakthrough in BBB-targeted neurological drug delivery, as illustrated in Figure 2. These particles, which are typically composed of polymers or metals, are capable of encapsulating the drug and targeting it to affected tissues via a magnetic field [67]. For instance, Kong and colleagues demonstrated that MNP can traverse normal BBBs using an external magnetic field in a mouse model. These MNPs are able to penetrate the BBB using an external magnetic field after systemic administration and accumulate in the perivascular region of the brain parenchyma [68]. Furthermore, penetrating peptides (e.g., angiopep-2 and Tat peptides) can bind to the MNPs, thereby enhancing their ability to across the BBB. These peptides are capable of binding to specific receptors on the BBB, thereby promoting the endocytosis of nanoparticles. The ability of MNPs to be manipulated in the presence of a magnetic field allows for their direction towards specific brain regions [69].

The dimensions of the magnetic nanoparticles, the magnitude of the external magnetic field, and the volume of blood flowing through the blood vessel all exert an influence on the permeability of the BBB. Gkountas and colleagues employed computational fluid dynamics (CFD) and discrete element methods (DEM) to simulate the blood flow and the behaviour of the MNPs in three dimensions and solved the Navier–Stokes equation. The findings demonstrate that the applied magnetic field facilitates enhanced drug delivery to the CNS [67].

The properties of magnetic nanoparticles can be modified according to the specific requirements of a given situation. The composition, size, and surface morphology of these nanoparticles can be tailored to enhance their ability. This approach can facilitate more effective treatment of neurological disorders [70]. In a recent study, Li and his research team proposed a novel platform combining the magnetic targeting and drug delivery properties of MNPs with the BBB-penetrating ability of engineered exosomes, which are capable of traversing the BBB and encapsulating small interfering RNAs (siRNAs). The platform is capable of enriching in the brain under localised magnetic localization, whereby membrane penetration is triggered by vasopeptide-modified engineered exosomes. This enables the particles to traverse the BBB and target GBM cells, enhancing iron death in the GBM. The platform exhibits good biocompatibility and safety [71]. Moreover, the Dai team employed iron oxide magnetic nanoparticles (IONPs) Fe_3_O_4_ as a carrier for the drug methotrexate (MTX), which was then guided to the tumor site via an external magnetic field. It was observed that this approach could promote apoptosis of DLBCL cells through the mitochondrial apoptosis pathway, offering a promising avenue for PCNSL treatment. The magnetically targeted thermo-chemotherapy approach has the potential to achieve superior therapeutic outcomes than single chemotherapy [72]. In conclusion, the utilization of MNP-targeted therapy for neurological disorders represents a promising avenue of research, yet further studies and clinical trials are required to ascertain its safety and feasibility.

### 4.2. Biological Methods

#### 4.2.1. Gene Therapy

Gene therapy is a technique whereby genetic factors are introduced into a patient’s cells with the objective of treating and preventing disease [73]. The process of gene therapy comprises three principal stages. The initial step is to identify the target disease, i.e., the disease for which gene therapy is considered as a potential treatment. Examples of target diseases include PD, AD, and spinal muscular atrophy (SMA), all of which are caused by mutations or abnormal expression of genes. Secondly, it is necessary to select vectors that can safely and efficiently deliver genes with therapeutic effects to target cells. At present, viral vectors and non-viral vectors (plasmid DNA, liposomes, etc.) are the vectors predominantly used in clinical practice. Ultimately, the therapeutic gene must be designed to meet the requisite specifications. In this process, it is crucial to consider not only the implantation of complete copies of genes into patients to replace or repair missing or mutated genes, but also the selection of genes that have specific roles. These are genes that regulate the expression of harmful mutations or provide the necessary repair in the event of damage to nerve tissue. This ensures that the therapeutic genes are customized and individualized, thereby maximizing the therapeutic effect. Vectors are a crucial component of gene therapy. They not only influence the efficacy of gene transfer but also determine the ultimate therapeutic outcome. Therefore, the selection of suitable and effective vector materials is of paramount importance to ensure the success of gene therapy. Vectors that can be utilized in gene therapy technology can be broadly classified into two categories: viral vectors and non-viral vectors.

##### Viral Vectors

Viral vectors, as a highly efficacious gene delivery tool, demonstrate considerable potential for traversing the BBB. By employing directed evolution techniques, scientists can identify viral variants that are more readily engulfed by BBB cells. This approach involves modifying viral vectors in a manner that emulates the co-evolutionary process observed between viruses and their hosts in nature, with the objective of enhancing their affinity and penetration ability with BBB cells. For example, through the targeted evolution platform CREATE (Cre-recombination-based AAV targeted evolution), researchers were able to identify capsid mutants capable of broadly transforming the central nervous system. These mutants exhibit a preference for vascular cells and astrocytes and target neurons with higher specificity across the blood–brain barrier in a variety of murine species [74].

Modifications to viral vectors can facilitate active transport and endocytosis by binding to specific transporter receptors on the BBB, thereby increasing their efficiency in crossing the BBB [75]. The efficiency and specificity of viral vectors for traversing the BBB can be enhanced through the engineering of the viral capsid. For instance, modifications to the surface proteins of adeno-associated virus (AAV) vectors can enhance their capacity to traverse the BBB, altering their structure and improving their interaction with BBB cells. Researchers have developed novel vectors that are capable of specific and efficient transduction into brain endothelial cells. One such vector is AAV-X1, which has demonstrated broad CNS targeting in rodents and non-human primates [76].

In conclusion, viral vectors can traverse the BBB in a number of ways. First, viral variants that are more susceptible to phagocytosis by BBB cells should be identified through directed evolution techniques. Second, viral vectors can be modified to bind specific transporter receptors, thereby facilitating their crossing of the BBB. The viral capsid can be engineered to improve its efficiency and specificity in crossing the BBB. The development of these strategies has provided new avenues for the treatment of CNS disorders, particularly in the field of gene therapy, where advances in these technologies are anticipated to result in the creation of safer and more effective therapeutic options. As research progresses, it is possible that new and innovative approaches may be developed in the future to overcome the limitations of the BBB for drug delivery.

##### Non-Viral Vectors

Non-viral vectors represent a significant advancement in gene transfection technology [77], particularly within the domain of gene therapy. These vectors are typically composed of either man-made or naturally occurring substances and are capable of delivering therapeutic genes to target cells in a safe and efficient manner. Non-viral vectors have low immunogenicity, favorable biocompatibility, capacity to transport substantial DNA segments, and simplicity of batch preparation [78]. Among these, plasmids, liposomes, and polymers are the most commonly utilized non-viral vectors [79]. The use of plasmid DNA as a vector is a relatively straightforward and cost-effective approach; however, the transfection efficiency of plasmid DNA is typically low, particularly in cells that are not dividing, which significantly constrains its applicability in the treatment of neurological disorders. The use of liposomes as carriers has the potential to facilitate the precise treatment of neurological diseases, given their favourable biocompatibility and high transfection efficiency. Polymeric materials (e.g., polyethyleneimine, poly-lysine, etc.) have the capacity to form stable complexes with drugs and genes, thereby facilitating their phagocytosis in cells.

The utilization of non-viral vectors in the treatment of neurological diseases is becoming increasingly prevalent. Some research groups have now prepared PPTA/pVAXI-En and conducted experimental verification of the exceptional characteristics of the PPTA/pVAXI-En complex in targeted transport [80]. This complex has been demonstrated to be capable of efficiently crossing biological barriers in the brain, specifically the BBB and the cerebrospinal fluid barrier (BTB), thereby achieving precise targeting of gliomas. In light of these findings, PPTA/pVAXI-En can be considered an innovative and multifunctional non-viral gene delivery system that enables direct delivery of gene therapy to the patient, offering a novel and highly efficient approach to neuroma treatment. Despite the current limitations of non-viral vectors in terms of transfection efficiency and targeting, the potential of their application in the field of gene therapy is gradually being explored and optimized.

In conclusion, gene therapy represents a highly promising new avenue of treatment for neurological diseases. RNA interference therapy has been demonstrated to impede the progression of Alzheimer’s disease by reducing the amount of toxic amyloid [81]. The utilization of gene editing technology for the treatment of Huntington’s disease has instilled optimism regarding the potential for direct repair of genetic defects [82]. While some of the aforementioned gene therapies have circumvented the challenge of directly penetrating the BBB, the delivery modes and mechanisms of action in these instances nonetheless offer valuable insights into potential strategies for blood–brain barrier penetration. For diseases that require crossing the BBB, the ventricular system or the transient opening of the BBB in pathological states may be viable routes for delivering therapeutic molecules. The selection of vectors, optimization of drug delivery modes, and design of therapeutic molecules are also crucial for the development of new strategies for blood–brain barrier penetration.

#### 4.2.2. Bioengineering Methods

##### Cell-Penetrating Peptide

**Figure 3:**
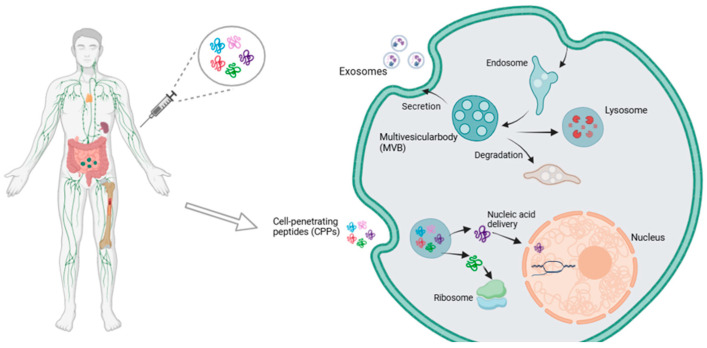
Schematic representation of the role of exosomes and cell-penetrating peptides in cells.

Cell-penetrating peptides (CPPs) [83] are short peptides that possess the ability to traverse cellular and tissue barriers, facilitating the intracellular delivery of biomolecules (e.g., proteins, nucleic acids, nanoparticles, etc.) [84] via endocytosis or direct penetration (Figure 3). CPPs offer several advantages over other unnatural small molecule chemicals. These include low toxicity to cells, rapid internalization properties, direct fusion with active proteins, and recombinant expression [85].

The endocytosis of cell-penetrating peptides is dependent on intracellular “molecular switches”, specifically RhoGTPases [86]. CPPs deliver peptide chains and their cargoes into the cell by activating the intracellular RhoA signaling pathway and forming endocytosis vesicles [87]. The direct penetration pathway is a non-energy-dependent crossing of the cell membrane that is effective at low temperatures [88]. The penetration efficiency of CPPs is influenced by alterations in cell membrane composition, specifically changes in cholesterol content, resulting from interactions with positive and negative charges [89]. It can be reasonably deduced that cationic CPPs are more likely to directly penetrate the cell membrane, given the evidence presented thus far. The direct traversal modes can be classified into three theoretical models: pore channel, transient pore channel, and trans-glue cluster. These models primarily function through the interaction of CPPs with the cell membrane, resulting in membrane perturbation and alterations in permeability [90]. Furthermore, arginine facilitates cellular membrane penetration [91]. The guanidine groups of CPPs, fatty acids, and the pH value of the cell membrane can also influence the mechanism of membrane penetration [92,93,94]. It is important to note that the transmembrane process is influenced by a multitude of factors, including peptide concentration, temperature, and cell type. Even a single membrane-penetrating peptide may alter its membrane-penetrating mechanism when environmental conditions are altered [95]. Furthermore, existing studies have indicated that the ability of cell-penetrating peptides to cross the BBB is not significantly linked to their cell penetration. In a study by Spiegeleer, five peptides were evaluated, and it was found that pVEC, SynB3, and Tat47-57 were able to enter the brain faster, while TP10 and TP10-2 had slower brain influx. The use of radiolabeled BSA as a marker failed to detect any presence in the brain, thereby ruling out the possibility of an increased permeability of the BBB [96].

Following the identification of the initial membrane-penetrating peptide, TAT, the process of membrane-penetrating peptide transport within the cell was subjected to rigorous examination. Subsequently, in vivo tests in mice demonstrated that TAT-coupled β-galactose-binding protein could enter the entire body of mice, including the brain, via intraperitoneal administration [97]. In recent years, research on penetrating peptides has yielded significant advances in the field of neurobiology, particularly in the areas of BBB disruption and the treatment of neurological disorders. Bioasis Technologies, a Canadian company, has developed a platform for crossing the BBB that employs a membrane-penetrating peptide, xB3 [98]. The platform achieves effective delivery of siRNA to the brain through the coupling of the peptide with small interfering RNA using receptor-mediated endocytosis, thereby opening up new pathways for the treatment of diseases such as stroke [99]. This provides innovative strategies for drug development. In a recent study, Habashi et al. [100] discovered two azapeptides [azaNle^3^]-1 (4) and [azaHse^6^]-1 (7) by replacing the amino acid residues in cyclic D,L-α-peptide with azaamino acids, which can effectively inhibit Aβ aggregation and alleviate symptoms in the Alzheimer’s disease model, providing a new idea for the treatment of Alzheimer’s disease.

##### Exosomes

Exosomes are membranous vesicles that are released into the extracellular matrix through the fusion of intracellular multivesicular bodies (MVBs) with cell membranes. They have a diameter of approximately 30–100 nm and possess a distinctive membrane structure, as well as a high concentration of bioactive components [101]. Exosomes demonstrate considerable potential for use in targeted drug delivery strategies across the BBB. They are capable of entering target cells via receptor-mediated endocytosis or direct membrane fusion, effectively circumventing the BBB and achieving precise drug delivery [102]. The mechanism of exosome action is illustrated in Figure 3. Furthermore, the biocompatibility and low immunogenicity of exosomes mitigate the risk of adverse effects during treatment.

The research team led by Prof. Dong-Gyu Jo has developed a novel therapeutic protein delivery technology, which they have designated as MAPLEX [103]. This technology is based on the natural membrane-penetrating properties of exosomes and their low immunogenicity, and it transforms exosomes into a nanoscale biomolecule delivery vehicle. Additionally, the team has constructed a light-controlled drug release apparatus to ensure the precise release of therapeutic proteins to target cells. The team plans to further optimize the MAPLEX system and explore its potential for use in the treatment of rare genetic diseases. In a separate study, Wang and colleagues explored the potential of 6-hydroxydopamine to create a model of Parkinson’s disease [104]. They discovered that human umbilical cord mesenchymal stem cell-derived exosomes loaded with brain-derived neurotrophic factor could serve as an effective drug delivery platform. This approach was observed to significantly inhibit apoptosis and enhance neuronal survival through the BBB. The new strategy for PD treatment is based on the ability of the drug to regulate the expression of phosphorylated tau and microtubule-associated protein 2, as well as enhance cellular antioxidant defense mechanisms.

##### Stem Cell Delivery

Stem cells are a class of cells that possess the capacity for self-renewal and multidirectional differentiation [105]. There is considerable evidence that stem cells can play an important role in the treatment of neurological diseases. Induced pluripotent stem cells (iPSCs) can be induced to differentiate into specific cell types (e.g., neurons), thereby providing a viable pathway to specific therapies for PD [106,107]. By means of gene editing or transfection technology, Mesenchymal stem cells (MSCs) can be modified to express or carry specific drugs or therapeutic genes, thereby enabling the direct injection or crossing of the BBB to facilitate the delivery of therapeutic substances to the interior of the brain or the site of tissue damage within the brain. It is anticipated that this drug delivery method will facilitate the development of novel strategies for the prevention and treatment of neurodegenerative diseases.

MSCs possess a natural homing capacity, whereby they respond to inflammatory signals at the site of the lesion, thereby guiding their migration to the lesion or damaged area [108]. During the guidance process, MSCs can traverse the endothelium through processes such as adhesion of integrins to vascular endothelial cells and regulation of endothelial cell tight junctions [109]. Furthermore, MSCs are capable of communicating with host cells and regulating the body’s immune response through the secretion of cytokines, growth factors, and miRNAs [110], which facilitates the survival and functioning of MSCs within the target organ, thereby enhancing their effectiveness as a drug delivery system. The homing process of MSCs can be either systematic or non-systematic [111]. Non-systematic homing refers to the targeted migration of MSCs to the damaged area for therapeutic purposes via pathways such as chemokines. In contrast, in the case of systemic homing, the location at which the MSCs are administered is situated at a greater distance from the lesion. In the context of drug-targeted delivery, target genes can be inserted into the MSC genome through genetic engineering to ensure the continuous secretion of therapeutic proteins after reaching the target tissues. Alternatively, MSCs can be combined with drugs and nanoparticles to create a composite, thereby enhancing the safety and efficacy of the MSC delivery system [112,113]. Clinical trials have demonstrated that these therapies are relatively safe, with no serious adverse effects reported [114].

#### 4.2.3. Receptor-Mediated Delivery

Targeted receptors on the BBB are specific proteins located on the endothelial cells of the brain’s microvasculature that facilitate the targeted entry of drugs or other molecules into the brain [115,116,117,118].

The transferrin receptor (TfR) is a receptor that is highly expressed in brain capillary endothelial cells of the BBB and is essential for brain iron metabolism and function [119]. The research team led by Bourassa discovered that the function of the TfR within the BBB remained unaltered in the context of AD. Furthermore, their findings indicated that the protein levels and internalization mechanisms of TfR were not influenced by the neuropathology of amyloid-beta (Aβ) and tau. This suggests that TfR may be a potential target for drug delivery to brain capillary endothelial cells, even in AD [119]. Barker et al. employed an oligonucleotide transfer vector (OTV), incorporating human transferrin receptor 1 (TfR1), to facilitate the delivery of antiviral oligonucleotides (ASOs) into the CNS. The intravenous injection of OTV in transferrin knock-in mice (TfRmu/hu KI) and rhesus monkeys demonstrated that OTV was capable of efficiently crossing the BBB and achieving a uniform distribution of ASOs in the CNS, exhibiting a silencing effect on target RNAs. This novel platform may offer an efficacious strategy for ASO delivery, with the potential for clinical translation [120].

Insulin receptor is a transmembrane dimeric protein comprising two subunits. It activates tyrosine kinases by binding to insulin, thereby initiating intracellular signaling pathways [121,122,123]. The presence of IR on the BBB allows drugs to be effectively delivered to the brain via its mediated transcytosis [124]. For instance, to address the inability of recombinant IDUA to cross the BBB in mucopolysaccharide storage disorder type I (MPS I), Giugliani’s research team redesigned IDUA as a trans-cerebral insulin receptor antibody-aiduronidase fusion protein. Following a phase 1–2 clinical trial of the trans-brain insulin receptor antibody-aiduronidase fusion protein administered intravenously to pediatric patients with MPS I, the results demonstrated that the treatment stabilized CNS function and somatic symptoms, indicating the potential for utilizing the insulin receptor to achieve BBB crossing [125].

CD98 heavy chain (CD98hc) is a cell surface transmembrane glycoprotein encoded by the SLC3A2 gene. CD98 can influence processes such as cell proliferation and migration [126,127,128]. CD98hc is also a target receptor for BBB transport, and substances can be transported across the BBB via its mediated transcellular transport (RMT) [116]. Chew’s team developed ATVCD98hc, an antibody transporter that specifically binds CD98hc, by modifying an IgG1 antibody, and evaluated its pharmacokinetics and biodistribution in model animals. The findings demonstrated that ATVCD98hc exhibited superior brain delivery compared to the TfR-targeted platform, with high brain concentrations and prolonged persistence observed in a humanized mouse model. These studies indicate that TVCD98hc has the potential to be a promising novel drug delivery platform across the BBB [129]. The study of these strategies has the potential to enhance the efficiency of BBB spanning, as well as to improve its safety and biocompatibility.

#### 4.2.4. “Trojan Horse” Strategy

The Trojan horse strategy represents a method for the delivery of macromolecular compounds, including proteins, DNA, siRNAs, and drug carriers, to the brain. This strategy is based on the concept of the Trojan horse as described in ancient Greek mythology [130]. The method employs brain transport vectors, including endogenous proteins and peptides, modified proteins and peptides that mimic monoclonal antibodies, and other molecules capable of targeting specific receptors or transporter systems. These vectors facilitate the crossing of the BBB via receptor-mediated transcellular transport [131]. Furthermore, drugs can be packaged with cell-penetrating peptides, various antibodies, modified proteins, exosomes, and other means of packaging to achieve BBB-targeted penetration strategies, which have been previously mentioned and will not be discussed in detail here.

As a “Trojan horse” delivery system, cells and cell-derived vesicles have great potential in diagnosing and treating brain diseases, with high biocompatibility, low immunogenicity, specific tissue homing ability and the ability to penetrate BBB [132]. A variety of stem cells and immune cells can be utilized as drug carriers, in addition to T-cells, which are capable of traversing the BBB and migrating to inflamed lesions [133] and aggressive brain tumors [134]. Ayer’s team synthesized PEG-modified polystyrene nanoparticles with maleimide groups and covalently bound them to T-cells. In mice that had been pretreated with TNF-α, the researchers evaluated the capacity of these modified T cells to transport the nanoparticles to the CNS. The findings demonstrated that these T cells were capable of delivering nanoparticles into the mouse brain, indicating the potential for T cell subsets to facilitate brain delivery in a xenogeneic host and offering new avenues for the utilization of immune cells as drug delivery vehicles across the BBB [135].

Furthermore, the BBB can be crossed by encapsulating drug-carrying nanoparticles or drugs using cell membranes [136]. The core contains the drug, while the outer membrane includes proteins that interact with receptors on BBB cells, enabling BBB targeting across the BBB. The glioblastoma therapeutic nanoparticles, which are based on elevated levels of lactate and encapsulated in glioma cell membranes, were able to readily cross the BBB and target the GBM. They demonstrated strong anti-tumor effects through lactate metabolism and photodynamic therapy in ex vivo models. This offers a novel approach to GBM treatment, while demonstrating the potential to enhance therapeutic efficacy by leveraging the metabolic characteristics of the tumor [137].

### 4.3. Chemical Method

The utilization of chemical modifications to enhance the lipophilicity of a pharmaceutical agent and facilitate its traversal across the BBB represents a promising avenue for a multitude of potential applications [138]. Prodrug design can enhance the physicochemical properties of a drug through chemical modification, thereby increasing its solubility and bioavailability. This approach is particularly effective for drugs that otherwise have poor solubility and low bioavailability [139]. Chemical modification offers numerous advantages. By enabling targeted delivery of a drug through chemical modification, the risk of damage to normal tissues is reduced. Moreover, chemically modified drugs with specific functions can be designed for specific diseases, thereby facilitating personalized therapy.

#### 4.3.1. Chemical Modification

The objective of chemical modification techniques is to enhance the molecular properties of drugs, thereby facilitating their crossing of the BBB. One of the key principles is to augment the lipophilicity of a drug through chemical modification, such as the introduction of lipophilic moieties, to facilitate its passage across the BBB. Lipophilic drugs are capable of more effective interaction with the lipid bilayer of the BBB, thus facilitating its passage. This entails the chemical modification of the drug into a prodrug, as well as the enhancement of its permeability across the BBB by means of an increase in the drug’s lipophilicity [138]. For instance, peptide drugs are not readily permeable across the BBB due to their high water solubility. Consequently, they must undergo chemical modification to enhance their lipophilicity, thus improving their delivery efficiency in the brain [140].

Furthermore, the physicochemical properties of drug molecules can be optimized through the reduction of hydrogen bond donors, the deletion or replacement of negatively charged atoms to reduce the surface area charge density (tPSA), the removal of basic groups to reduce pKa, and the introduction of constrained conformations to enhance molecular rigidity, thereby improving their BBB permeability and reducing the rate of exocytosis [141]. Nanomaterials can be delivered into the body circulation after drug administration and may be distributed to various organelles, including the brain. Chemically modified nanomaterials have the potential to interact with cell membranes, enter cells, and modulate a variety of biological processes, thereby enhancing drug delivery efficiency [142]. Chemical modification plays an indispensable role in the treatment of CNS diseases.

#### 4.3.2. Prodrug Strategies

The prodrug strategy represents a well-established technique in the field of drug design, whereby an active drug (the prodrug) is chemically modified into a relatively inactive or completely inactive form prior to administration. It is estimated that approximately 5–7% of all globally approved drugs can be classified as prodrugs [143]. The fundamental objective of the prodrug approach is to optimize the physicochemical properties of a drug in order to enhance its therapeutic performance. Prodrug design facilitates enhanced solubility, augmented stability, and diminished toxicity, thereby rendering drugs more suitable for clinical application. Furthermore, prodrugs can be designed to target specific tissues or cells by incorporating chemical modifications that facilitate localized drug release. This selective targeting not only increases the concentration of the drug at the desired site but also reduces adverse effects in non-target tissues.

One of the most significant advantages of the prodrug approach is its capacity to regulate the release of the active pharmaceutical agent in response to specific physiological stimuli. Moreover, the prodrug strategy can enhance oral drug absorption and systemic bioavailability by circumventing hepatic first-pass metabolism, thus augmenting the therapeutic efficacy of orally administered drugs [144,145].

The use of prodrug strategies in clinical practice is widespread and covers a wide range of drugs and therapeutic areas. Levodopa for the treatment of PD is a prodrug of dopamine that can cross the BBB and be converted to dopamine in the brain, thereby effectively treating Parkinson’s. This prodrug strategy allows the drug to reach the brain more efficiently and exert its therapeutic effects [146]. The prodrug strategy can improve drug targeting by designing specific chemical structures that allow the drug to remain inert when it reaches the BBB and to be activated by specific enzymes or conditions once it enters the brain, thereby increasing the concentration and efficacy of the drug in the brain. For example, the strategy of internal activation of prodrugs using features of the tumor microenvironment (e.g., abnormal redox environment, acidity, hypoxia, enzymes, etc.) has been used in cancer therapy [147]. The prodrug strategy can improve the physicochemical properties of the drug, such as by increasing the water or lipid solubility, increasing the absorption of the drug for oral or topical administration, and thus improving the bioavailability of the drug. This is particularly important for BBB delivery, where drug permeability is severely limited. In addition, prodrug strategies can enhance drug penetration across the BBB by designing prodrugs that can be recognized by specific transporter proteins on the BBB, thereby exploiting the natural functions of these transporter proteins. This provides a new perspective on BBB drug delivery, and advances in prodrug technology are expected to lead to safer and more effective therapeutic options.

### 4.4. Nanoparticle Delivery Systems

A nanoparticle delivery system represents an advanced drug delivery platform developed through nanotechnology. This system encapsulates or adsorbs drugs into nanoscale particles. The BBB is highly selective, restricting the entry of macromolecules and most small-molecule drugs, including those used to treat neurological disorders, thereby compromising the efficacy of drug therapies.

Nutrients are transported into the brain via solute carrier (SLC) proteins expressed on the BBB [148], and this mechanism can be exploited to enhance the delivery efficiency of drugs encapsulated in nanoparticles. Specifically, ligands that mimic substrates of the carrier proteins on the BBB—such as glucose or amino acids—can be selected [149,150] and chemically modified onto the surface of nanoparticle carriers. By leveraging this approach, the nanocarriers can cross the BBB through interactions with specific binding sites on the carrier proteins, allowing for more targeted delivery of drugs.

It is critical to consider two primary factors in selecting suitable substrates for modification. First, the substrate should have high expression levels of the corresponding SLCs on the BBB, to maximize the chances of effective transport. Second, there must be an appropriate affinity between the substrate and the carrier proteins. An excessively high affinity may lead to the drug-nanocarrier complex being sequestered by lysosomes, which can prevent the drug from being released into brain tissue [151]. Therefore, achieving the right balance in substrate selection is essential to ensure the drug delivery system can traverse the BBB effectively without compromising the subsequent release of the drug.

One of the significant challenges associated with nanoparticle (NP) drug delivery systems is the inherent instability and potential toxicity of inorganic NPs. To address these concerns, surface modification techniques are often employed, wherein the NP surface is coated with biocompatible materials or surfactants. This approach, known as coating-encapsulation, stabilizes nanoparticles and improves their compatibility within biological systems, while simultaneously reducing systemic toxicity. Recent research has identified various materials as optimal surface coatings for nanoparticle delivery systems, such as PS80 [152], PEG [153], PLGA [154], and chitosan [155,156], each offering distinct benefits in terms of stability and biocompatibility.

In addition to surface modification, several physicochemical characteristics of NPs—such as size, shape, composition, and stiffness—play critical roles in determining their overall functionality [157]. These properties must be finely tuned to optimize both the stability of the nanoparticles and their interactions with the biological environment. Since the advent of nano/micron drug delivery systems over 50 years ago [158], the field has witnessed significant advancements. Today, a wide variety of nanostructured drug carriers have been developed for the treatment of neurological disorders.

In summary, nanoparticle delivery systems have shown great promise in improving the delivery of drugs across the BBB, providing a more targeted and efficient treatment for neurological disorders. Recent research has significantly advanced our understanding of how nanoparticle characteristics and surface modifications affect their ability to cross the BBB, offering insights into the development of safer and more effective therapeutic strategies. A comprehensive summary of current research into nanoparticle-based treatments for neurological disorders is presented in the following table (Table 1), highlighting the types of disorders, the nanoparticles used, their mechanisms of action, and the progress of ongoing research efforts. This compilation aims to provide a clearer picture of the potential and challenges of nanotechnology in the treatment of brain diseases.

#### 4.4.1. Polymer Nanoparticles

The objective of polymer nanoparticle delivery technology, which utilizes polymer materials to create nanoscale particles, is to enable the efficient and safe delivery of drugs. Selecting an appropriate polymer is crucial in this technology. The polymers used can be either natural or synthetic macromolecules. Natural macromolecules include proteins, peptides, and polysaccharides, while synthetic macromolecules include poly(lactic-co-glycolic acid) (PLGA), poly(vinyl alcohol) (PVA), and poly(styrene) (PS). The choice of polymer depends on the intended application and the specific performance requirements of the nanoparticles. Ideal polymers should be biocompatible [161], biodegradable, and easily functionalized [162]. Polymers play several roles in nanoparticle preparation, including imparting structural stability, modulating drug release, enhancing biocompatibility, improving nanoparticle stability and dispersion, and ensuring effective drug encapsulation and targeted delivery [163].

PLGA is a biodegradable polymer that is widely used in the preparation of polymer nanoparticles. The FDA has approved the use of PLGA in medical products [164]. Hu et al. [165] constructed a novel biodegradable cerebral drug delivery system, which was developed by coupling lactoferrin (Lf) with PEG–PLGA nanoparticles coupled to form Lf-NP. In zoological experiments, Lf-NP loaded with 6-OHDA was significantly attenuated 6-OHDA-induced striatal lesions, indicating the potential of this approach for the treatment of PD. The team led by Dr Nitin Joshi [166] employed PLGA to facilitate the penetration of siRNA molecules through the intact BBB of healthy mice in the course of their zoological experiments. The resulting data indicated a 50% reduction in tau protein expression in mice utilising the PLGA transport pathway in comparison to the control group. This finding suggests that this technology may have potential for use in clinical AD therapy. Furthermore, Katila’s team [167] employed a modified PLGA to facilitate the coupling of resveratrol (RSV) to create Lf–RSV–PLGA–NPs, which were then tested in vivo using a mouse model of MPTP-induced PD. The experimental results demonstrated that these nano-preparations possess enhanced neuroprotective properties and have promising clinical applications.

Furthermore, fucoidan nanoparticles targeting P-selectin represent an innovative polymeric nanoparticle drug-delivery platform designed to overcome the limitations of the BBB and enhance drug efficacy in treating brain diseases. The development of these nanoparticles is rooted in a comprehensive understanding of medulloblastoma (MB) [168], the most common intracranial neoplasm in pediatric patients. The challenges posed by the BBB contribute to a low cure rate and poor prognosis for MB. To address this issue, researchers [117] constructed fucoidan nanoparticles that target P-selectin by leveraging the mechanism whereby P-selectin, expressed by brain tumor vascular endothelial cells, traverses the BBB via caveolin-1-mediated transcytosis. This carrier system is utilized to deliver the anticancer drug Vismodegib directly to brain neural tissues, minimizing damage to surrounding normal brain tissues. Notably, this approach exhibits low toxicity, which is crucial for patients undergoing long-term anticancer treatment. The system holds promise for application in the treatment of various conditions, including brain tumors in both children and adults, epilepsy, multiple sclerosis, strokes, neurological lesions, and other related diseases.

Despite the considerable promise of polymer nanoparticle technology, the field still confronts a number of significant challenges. These include the precise control of particle size during production, ensuring stability in mass production, maintaining stability in long-term storage, and guaranteeing biosafety [169]. The resolution of these issues will facilitate the advancement of polymer nanoparticle delivery technology, enhancing its efficiency and safety.

#### 4.4.2. Liposome Nanoparticles

A liposome is a closed-loop vesicle comprising amphiphilic phospholipid bilayers with an internal aqueous cavity structure, which can be utilized to carry water-soluble drugs or molecules [170]. The concept of liposomes was first introduced by Bangham [171], and researchers have been working on liposome-encapsulated drugs to penetrate the BBB due to the favourable biocompatibility and biodegradability of liposomes. It was found that simple liposomes, although able to achieve targeting, were highly susceptible to being recognized and cleared by vascular endothelial cells, resulting in a short circulation time of the drug in the bloodstream. Subsequently, the staff modified the surface of the liposomes through PEGylation, thereby prolonging their time in the blood circulation. This resulted in the creation of long-circulating liposomes, also known as stealth liposomes [172]. An example of a long-circulating liposome is the Adriamycin liposome injection, also known as Doxil [173,174]. In recent years, liposomes have been used in the treatment of some neurological diseases. Senapati et al. [175] developed a multifunctional liposome platform that targets toxic amyloid β oligomers (AβOs) in Alzheimer’s disease by binding a self-assembled cyclic D,L-α-peptide (CP-2). These findings highlight CP-2-LPs as a promising diagnostic and therapeutic tool for early detection and targeted therapy of AD.

In view of the clinical requirements of diseases such as oncology, it is essential that carriers be capable of targeting liposomes with greater precision in order to minimize the toxic effects at unintended sites throughout the body [170]. From this, it can be seen that targeting of liposomes can be classified into two categories: active and passive. Passive targeting is unable to distinguish between intended and unintended cells; in contrast [176], active targeting, such as cell-specific targeting, enhances liposome targeting by incorporating a molecular recognition component [177]. An example of an active targeting drug is siRNA drug Onpattro (patisiran cationic liposome injections), which was developed by Alnylam [178] and has been successful in achieving nucleic acid drug delivery for tumor inhibition.

The advancement of nanocarrier technology has led to the emergence of microenvironment-sensitive liposomes [179], exemplified by ThermoDox (Adriamycin) [180], a heat-sensitive liposome developed by Celsion. While there is currently no evidence of the use of ThermoDox to cross the BBB for the treatment of neurological diseases, as a heat-sensitive liposome its capacity to penetrate the BBB can be enhanced by modifying its physicochemical properties. The development of pH-sensitive liposomes may be achieved through the modification of liposomes with pH-sensitive materials, in accordance with the pH differential between the lesion site (e.g., the BBB) and normal tissues. This approach enables the delivery of drugs in a targeted manner, based on the pH gradient. For example, Gong et al. [181] designed and used pH-sensitive liposomes (PSL-FTY720/AB) as a drug delivery system, achieving targeted delivery to the BBB in a brain hemorrhage model. Following a cerebral hemorrhage, a reduction in the extracellular pH of the region surrounding the hematoma resulted in the release of the drug from the nanoparticles. PSL-FTY720/AB was able to penetrate the BBB and accumulate in the hematoma and its surrounding region. This strategy not only increased the concentration of the drug in the brain lesion area, but also reduced the incidence of systemic side effects, thereby providing a new strategy for the treatment of neurological diseases.

Bionic liposomes represent a novel class of liposomes that have been developed on the basis of traditional liposomes and targeted liposomes. The latter are composed of biocompatible materials, including natural phospholipids, which are capable of mimicking the structure and function of biological membranes and are compatible with the in vivo microenvironment [182]. With regard to the treatment of neurological diseases, the advent of this technology offers a novel approach to circumventing the BBB. Han et al. [183] have synthesized a property-mimicking apoptotic erythrocyte bio-nano-mimetic liposome (Effero-RLP), which has been employed to enhance macrophage targeting and achieve targeted drug delivery. In the context of neurological disease treatment, the potential of this technology to facilitate targeted delivery is particularly noteworthy. The design of liposomes that can specifically bind to the BBB allows the drug to penetrate the BBB efficiently and increase the drug concentration in the diseased areas of the brain, thereby improving the therapeutic efficacy and reducing systemic side effects. It is also noteworthy that bionic liposomes have the capacity to mimic different receptors and channel proteins on the cell membrane [184,185], thereby offering a novel avenue for drug delivery targeting the BBB.

Solid lipid nanoparticles (SLNs) [186] represent a promising therapeutic tool for targeted drug delivery, with the potential to overcome the barriers posed by the BBB through the modification of targeted ligands on their surface. Furthermore, SLNs can be administered in a specific and controlled manner, thereby reducing the potential for toxicity issues. SLNs can be used as carriers for CNS diseases, such as AD [187], PD [188], HD [189], MS [190], epilepsy [191], ischemic stroke [192], brain tumors [193], and cancers. This technique is well-targeted and safe, with low toxicity and side effects, and a long drug half-life. The advantages of this technology are evident if it is applied in a clinical setting. Nevertheless, SLNs exhibit limited drug loading capacity, intricate liposome morphology, and instability issues, including gelation, particle enlargement, and drug release during transport [194]. Although the existing SLN targeting therapy for neurological diseases is still in the research stage, it is anticipated that it will become a new targeting drug once the transmembrane transport mechanism of the BBB has been fully elucidated.

A polymer-locked membrane fusion liposome was developed jointly by Zhao and Cai’s team [195]. The liposome encapsulates siRNA or CRISPR-Cas9 polymers into liposomes by means of a non-traceable ROS shearable linker strand, so that fusion only occurs after it crosses the BBB to reach the GBM tissues, which are high in ROS content. Further research is required to optimize the design of liposomal nanoparticles in order to enhance their targeting and efficacy, while ensuring safety and regulatory compliance, with a view to advancing the use of liposomal nanoparticles in clinical practice.

#### 4.4.3. Polymer Micelles

Polymer micelles (PM) are colloids formed by the self-assembly of amphiphilic polymers with one or more cores and unique properties. In solution, below the critical micelle concentration (CMC), these polymers exist as single molecules. However, above the CMC, they self-assemble into micelles, forming an inner lipophilic core and an outer hydrophilic shell [196]. This structure enables polymeric micelles to effectively encapsulate hydrophilic or hydrophobic drug molecules. The smaller size of polymeric micelles allows for more effective passive targeting of solid tumors and promotes cellular internalization in comparison to other nanocarriers [197]. The lipophilic core of these micelles is capable of effectively solubilizing hydrophobic compounds, while the hydrophilic corona has the effect of prolonging the circulation time of the micelles in the bloodstream [198]. Furthermore, polymer micelles are relatively straightforward to prepare and can be readily scaled up for production, rendering them more viable than polymer nanoparticles and liposomes, which necessitate intricate fabrication processes [199].

The internalization of PM within cells is primarily achieved through endocytosis, followed by cellular uptake and translocation to endosomes, and eventual release of the drug in the cytoplasm [200,201]. The release of drugs from PM can be achieved either by diffusion of the drug from the intact micelles or by breakdown of the micelles. The current limitations of clinical studies of PM are related to its stability, blood circulation time and controlled drug release. In light of these challenges, researchers have proposed enhancement of the stability and controlled release of PM, including increasing the length of the hydrophobic portion [202], functionalizing hydrophobic blocks and crosslinking the micellar core [203]. Furthermore, the stability of micelles must be evaluated under conditions that are relevant to biological systems, as proteins in plasma may form a protein corona, which could affect their biodistribution and cellular uptake [204]. Furthermore, it is crucial to examine stimuli-responsive polymeric micelles, which are distinguished by their intelligent responsiveness, reversibility, targeting and capacity to regulate drug release in response to biological or artificial stimuli [205,206,207].

A novel drug delivery system for trans-BBB has been designed by Ouyang and colleagues at Shenzhen University. Lf-PIC@Se polymeric micelles [208] were synthesized using an optimized method and modified at the surface, resulting in highly efficient drug loading and a stable micellar structure. In vitro experiments demonstrated that Lf-PIC@Se micelles exhibited excellent stability and enhanced cellular uptake efficiency through specific receptor-mediated mechanisms. Furthermore, these micelles have antioxidant effects that protect neuronal cells, thereby opening up new avenues for the treatment of CNS diseases. In another study, Chen’s group developed a novel polymeric micellar drug delivery system for acute ischemic stroke, which is primarily composed of reactive oxygen species’ (ROS) response and fibronectin-binding polymers [209]. The system is capable of traversing the compromised BBB by binding to microthrombi, which facilitates access to the ischemic region. In an oxidative stress microenvironment, the system is capable of releasing drugs that result in neuroprotection, modulation of microglia function, and restoration of BBB function.

### 4.5. Combinatorial Strategies

Table 2 is a summary of the different cross-BBB technology classifications mentioned above, which can help readers to better review the topic nd provide a summary reference for readers. Combinatorial strategies entail the utilization of multiple drug delivery technologies that interact and collaborate. These strategies encompass physical means, biotechnology, chemical pathways, or a combination of these approaches, and are designed to compensate for the shortcomings of a single technology in order to enhance drug distribution, bioavailability, and therapeutic efficacy in the brain.

#### 4.5.1. Enhanced Drug Delivery Based on Physiological Regulation

A research team from the School of Pharmacy at Southwest University has developed an innovative drug delivery system based on physiological modulation. This system enhances drug distribution in the brain and improves the likelihood of crossing the BBB by using perillylamine alkaloid-derived ionizable lipid molecules as drug carriers, along with selective modulation of cerebral microvascular blood flow [210]. This approach not only facilitates the penetration of drugs across the BBB but also enables precise intervention and synergistic treatment for specific brain diseases. It is anticipated that this strategy will open new avenues for the treatment of central nervous system diseases.

#### 4.5.2. Multimodal Delivery Systems

In 2013, Mura and colleagues provided a detailed analysis of the design principles and preparation methods of stimuli-responsive nanocarriers for drug delivery in their article, *Stimuli-responsive nanocarriers for drug delivery* [211]. They emphasized the importance of material selection and structural optimization, providing valuable insights into combinatorial strategies. In 2021, Wu et al. published a review in *Anticancer Agents in Medicinal Chemistry* [212], which introduced a novel dual-stimuli-responsive nanoparticle system. These nanoparticles respond to specific stimuli at particular times and locations, allowing for precise drug delivery and controlled release. This approach not only improves therapeutic efficacy but also reduces adverse effects associated with drug transport. In the same year, Jia et al. [213] integrated diverse stimuli-responsive properties, such as pH, temperature, and enzyme activity, to achieve precise drug release. The authors emphasized the importance of intelligent nanoparticles in overcoming obstacles to drug delivery by incorporating multiple response mechanisms.

This multimodal strategy is particularly significant for trans-BBB drug delivery. This increases drug concentration in the brain while minimizing systemic side effects. These advances in research provide new opportunities for the development of drug delivery systems capable of crossing the BBB. Additionally, different drugs or therapeutic molecules can be combined with nanoparticles to achieve synergistic treatments, improving overall therapeutic outcomes. Modifying the structure and surface of nanoparticles also allows for the customization of treatment strategies based on the specific requirements of various neurological disorders.

## 5. Summary and Prospects

The development of effective drug delivery strategies is critical to overcoming the major challenge posed by the BBB. As a result, researchers are continuously developing novel drug delivery technologies aimed at optimizing drug targeting in the brain while minimizing damage to non-target tissues. These innovative approaches have made significant progress in improving drug delivery efficiency and offer new hope for the treatment of CNS diseases.

This review highlights various strategies that have been developed to overcome the BBB, including physical, chemical, and biological approaches. For example, ultrasound-mediated BBB opening is a promising physical tool. It uses focused ultrasound and microbubble technology to temporarily and locally open the BBB, thereby increasing drug permeability. However, this technique may induce local inflammation or tissue damage, which requires further safety optimization. Magnetic targeting, another approach, uses magnetic nanoparticles to guide drugs to specific brain regions and offers excellent targeting potential. However, its efficacy depends on the strength and distribution, and there are technical challenges associated with deep brain targeting. Receptor-mediated delivery offers precise targeting by binding drugs to specific receptors on the BBB. While providing precise targeting, this method is limited by receptor expression levels and can induce immune responses or drug resistance. Polymer nanoparticle systems, on the other hand, offer excellent performance in terms of slow drug release, stability and BBB permeability. These systems allow precise control of the timing and location of drug release, but face challenges in terms of toxicity, long-term stability, and material degradation. Gene therapy represents an innovative approach with the potential to repair or replace diseased genes for long-term efficacy. However, it is currently limited by low delivery efficiency and immune rejection issues. CPPs and exosomes, both of which are biocompatible and capable of penetrating cells, hold promise as bio-delivery tools, but further research is needed to evaluate their in vivo stability and specificity. Meanwhile, stem cell delivery and smart dual-stimulus-responsive nanoparticle systems show potential for personalized therapy and response to environmental stimuli, respectively, but their complexity and high cost limit widespread clinical use. We summarize the different approaches to spanning the BBB, categorized by the latest technologies, and clarify the advantages of the different techniques (Table 2). We hope to provide a clear perspective and help readers better understand the linkages between these strategies.

Looking ahead, the development of multifunctional drug delivery systems is a prominent trend. These systems aim to integrate multiple functional modules to enable targeted delivery and controlled drug release. Smart-responsive drug delivery systems, such as nanomaterials that respond to light, pH, temperature or specific biomarkers, will also play a critical role in precision medicine. In addition, the development of novel biodegradable and biocompatible materials is expected to reduce toxicity and immunogenicity, thereby improving the safety and efficacy of drug delivery systems. Future research should focus on optimizing existing delivery technologies through further mechanistic studies. A deeper understanding of the varying BBB permeability in different patients could provide a basis for the development of personalized drug delivery strategies. In the context of neurological diseases, personalized delivery strategies can be designed according to the pathological characteristics of the patient and the unique properties of their BBB. This approach not only enhances drug efficacy but also reduces side effects, thereby advancing the field of precision medicine. In addition, the industrialization and regulatory oversight of drug delivery technologies are critical aspects that will determine their future success. It is essential to ensure that clinical translation and large-scale production of these systems meet stringent standards for safety, efficacy, and reproducibility [214,215].

In summary, while the advancement of drug delivery technologies offers innovative solutions for crossing the BBB, many technical and practical challenges remain. Nevertheless, these advances hold immense potential for improving the treatment of neurological diseases. As technology advances and personalized medicine continues to evolve, future drug delivery systems are expected to provide more precise and effective treatment options, ultimately improving therapeutic outcomes and quality of life for patients.

## Figures and Tables

**Table 1:** Application and safety analysis of nanoparticles in the treatment of different neurological disorders.

Disease Type	Type of Nanoparticles	Mechanism of Action	Research Progress	Security Analysis
Alzheimer’s Disease (AD)	Various nanoparticles (including organic and inorganic)	Increasing ACh levels, activating BDNF/TrkB signaling pathway	Donepezil and other drug nanoparticle forms have shown potential in clinical trials	Potential neurotoxicity of nanomaterials needs to be considered [142]
Parkinson’s Disease (PD)	Gold nanoparticles (AuNP)	Neuroprotective and anti-neuroinflammatory effects	AuNPs have shown therapeutic potential in cellular and animal models	The neuroprotective mechanisms of AuNPs are being intensively studied [159]
Stroke	Magnetic nanoparticles	Enhancing the efficacy of Transcranial Magnetic Stimulation (TMS)	Magnetic nanoparticles delivered via nasal spray enhance the effects of TMS	Non-invasive treatment method, further clinical validation is needed [160]
Brain Tumors	Various nanoparticles	Drug delivery, overcoming the blood–brain barrier	Nanoparticles used to increase the concentration of chemotherapy drugs in the brain	The biocompatibility and clearance mechanisms of nanoparticles are key [142]

**Table 2:** Different techniques for drug delivery across BBB.

Categorization	Technology Name		Basic Principle	Advantages
Physical Method	Ultrasonic Open BBB		Utilizes the mechanical effects of ultrasound to temporarily increase BBB permeability	Non-invasive, reversible, and allows for precise regional control
	Magnetic Targeted Delivery		Employs magnetic nanoparticles to achieve targeted drug delivery under an external magnetic field	Provides precise spatial control, minimizing damage to healthy tissues
Biological Methods	Gene Therapy	Viral Vectors	Leverages the natural ability of viruses to deliver genetic material into cells	Offers high efficiency in gene transfer, suitable for gene therapy applications
		Non-Viral Vectors	Utilizes non-viral carriers such as liposomes or polymers for genetic material delivery	Exhibits lower immunogenicity, safety, and scalability for production
	Bioengineering Methods	Cell-Penetrating Peptide	Deploys cell-penetrating peptides to facilitate the intracellular delivery of drugs or genes	Enhances intracellular drug delivery efficiency, potentially improving therapeutic outcomes
		Exosomes	Harnesses naturally secreted exosomes as carriers for drug or genetic material	Benefits from natural biocompatibility, avoiding immune system recognition and clearance
		Stem Cell Delivery	Utilizes stem cells as carriers for the delivery of drugs or genetic material	Capitalizes on the self-renewal and differentiation capabilities of stem cells, applicable to a range of therapeutic strategies
	Receptor-Mediated Delivery		Exploit the specific binding of cell surface receptors to drugs or carriers for targeted delivery	Improves drug targeting, thereby reducing side effects and enhancing therapeutic efficacy
	“Trojan Horse” Strategy		Exploits the cell’s natural uptake mechanisms to disguise drugs or carriers as substances normally internalized by cells	Enhances intracellular drug delivery efficiency, potentially leading to improved therapeutic effects
Chemical Method	Chemical Modification		Alters the physicochemical properties of drugs to enhance their ability to penetrate the BBB	Can improve drug bioavailability and therapeutic efficacy
	Pre-Drug Strategies		Converts active drugs into inactive or less active prodrugs, which are activated after delivery	Improves drug stability and bioavailability, potentially reducing side effects
Nanoparticle Delivery Systems	Polymer Nanoparticles		Employs polymeric nanoparticles as carriers for drug or genetic material delivery	Protects drugs from degradation and enhances stability and targeting capabilities
	Liposome Nanoparticles		Utilizes liposomes as carriers for drug or genetic material delivery	Offers biocompatibility and enhances drug stability and targeting
	Polymer Micelles		Uses polymeric micelles as carriers for drug or genetic material delivery	Improves drug solubility and bioavailability, potentially reducing side effects
